# Dissertation on the State of the Dental Profession, and Dental Empiricism

**Published:** 1845-09

**Authors:** James Taylor


### ARTICLE V.

*Dissertation on the State of the Dental Profession, and Dental Empiricism.* Delivered before the American Society of Dental Surgeons, at their Sixth Annual Meeting, held in the City of New York, August, 1845. By JAMES TAYLOR, D. D. S.

*Mr. President, and Gentlemen*
*of the American Society of Dental Surgeons:*

I CANNOT find language to express the gratification I derive from meeting so many of my professional brethren on this occasion. At any time, and under any circumstances whatever, an interview with those identified in pursuit, in feeling and interest, should be agreeable. What then should be our feelings, when thus, from all parts of our common country, we have assembled on an occasion like the present? and when the great object of our meeting together, is not only to form and renew acquaintance, to exchange views on scientific subjects, but also to do all in our power to advance, on a sure and firm basis, that particular branch of science in which we have embarked our energies, time and talents.

The pleasure of beholding, for the first time in my life, this great and growing city, has been rendered doubly interesting to me, because I, for the first time, also, meet with many of those whose names rush to memory's entablature, whenever early recollections—the bright spots of our existence—are dwelt upon.

This pleasure is also heightened by the fact, that I can now, by my presence, signify the deep interest I now feel, and *have always felt*, in the objects of this association. If you of the east can enjoy this rich interview of kindred feeling and professional courtesy, he who hails from the west has full as much to make it pleasant and agreeable, for he not only enjoys the same intercourse of thought and sentiment, but he forms acquaintance with those who have long been advancing up the hill of dental science, and who, from their kind demeanor on this occasion, appear not disposed to retard the onward progress of their brethren.

When I take a retrospect of the past, I can scarcely realise my present position. Time is certainly, in its rapid and fleeting course, stamping, with unerring accuracy, on all around us, change! change! The modes of travel change; steam cars, in their rapid flight, scarce allow a peep at the magnificent peaks of the Alleghanies—space is annihilated, and the Queen City of the West and the Emporium of the East are fast approaching; the great magnets fixed at both extremes, have, by the revolution of time, been brought to bear on each other: how soon the electric current may be so established, that we, like telegraphic despatches, may be made to pass from one pole to the other, I would scarce dare prophecy.

While thus around us change is stamped on the improvements of the age, this meeting reminds me that a change has come over the "spirit of our dream." Social intercourse is enlarged, the contracted, selfish feeling, which struggles to conceal all for its own benefit, is fast giving way to a more enlightened spirit; and, perhaps, in no profession has a greater change taken place, in this respect, than ours. Let us hope that this spirit will extend, until all shall vie with each other in disseminating the greatest amount of useful knowledge—until, at least in dentistry, none shall claim to alone possess the great secret of our art—until true merit alone shall claim its just reward; and all feeling of envy be swallowed up in the great effort to elevate the standard of dental excellence.

Was it appropriate on the present occasion, I might draw a pleasing contrast on the spirit which characterises the intercourse of the dental profession now and eighteen years ago: one was strictly the age of secret mystery, when not only knowledge was in danger of being feloniously taken, but dental instruments were considered really in danger of changing ownership, or imparting some useful knowledge. The other is an age of generous liberality, as this meeting, the kind hospitality which has met me at every turn of my journey, the readiness which has every where been displayed by my professional brethren, to exhibit their instruments and explain their mode of operating, abundantly prove.

Has not this association been instrumental in bringing about this change? where else can we look for a cause exerting so powerful an influence? True, all has not yet been accomplished—many errors yet prevail; yes, errors which never could be remedied single-handed, but which require united action, co-operative energy and mutual effort. Observation, since the formation of this society, impels me to make one remark, while on this subject, which is, that during the last few years, where I have met with a churlish, unsocial and illiberal dentist, he has not been an active member of this or any other dental association, for any length of time. I do not say that all who thus hold themselves aloof from the efforts now making to elevate dental science, are churlish and illiberal; some good operators, I doubt not, thus refuse to act, because they think no good can be effected; yet I do think, were they to observe the change already alluded to, that light would burst upon their vision, and they would behold our *dental bark* not so poorly manned as they imagine. I scarce can think that any worthy of the name of dentist are afraid of the strongest light of the meridian sun; many I know there are, claiming that appellation, who grope their way in dark and benighted paths, and are afraid that light will burst upon them, and expose all their deformity. I hope soon to see this number diminish, and all who view our vessel too rotten for repair, utterly desert her; we want no such hangers on to the sides of our craft, to disgrace her beautiful proportions, for she is a noble vessel, fit for any sea, safe in any storm, none too good to grace her decks, the noblest can find an honorable berth aboard, and rest secure beneath her outspread sails. What vessel rides more gallantly the onward wave to greatness; although often manned by an inexperienced crew, she falters not in her course; she has weathered storms from without and storms from within—the indiscreet blows of friends, and the insidious attacks of traitorous foes—yet with all these drawbacks, her fame is advancing, her utility increasing, and her worth more and more appreciated. What, let me ask, might have been her destiny, if always only a worthy crew had been aboard, and at her helm none but those worthy of so great an honor.

Dental science, thus trammelled and fettered by a host of unworthy pretenders, has continued to advance in importance, until her services have become indispensable to the health and comfort of nine-tenths of the community. Why, Mr. President, let me ask, do not all these avail themselves of the benefits to be derived from dental operations? The cause of dental science, our interest, and the public good, require that this question should be properly answered. Perhaps a few moments can be as well spent in investigating the cause, as in any other way.

What, then, is the cause? I answer, want of faith; and this want of faith is produced by the many imperfect and unskilful operations which disgrace our profession. I ask not for that mustard seed of faith necessary to remove a mountain, but simply that faith which is founded on the evidence of things seen, and less than is necessary to believe that every pretender is equally qualified with the most skilful, to discharge the duties of the profession—less than would induce a belief in the dental ligament, or that all dental operations can be effectual without producing any pain—only that faith which must believe that skill and care are necessary to preserve the dental organs. I take it for granted, that there is enough of that which is good, daily seen as the result of dental practice, to produce that faith in the mind of every careful observer. But we must recollect that one failure produces a greater impression than a dozen successful operations : a hundred cases might be given to illustrate this ; one case shall suffice. A village is visited by a dealer in dental notions—one of your real knowing chaps, who would not deign to spend more than three or four weeks in acquiring any profession : his first case is an aching tooth ; this he plugs, instead of extracting ; the pain is increased, and the whole village sees the swollen, distorted countenance of their neighbor, and he, in his agony, exclaims, "it is the last tooth I'll ever have plugged ; away with your dentistry, it is a humbug." Not one effort has been made to ascertain the qualifications of the operator, or the cause why the operation should not be of service, but the whole profession is viewed as being on a par with this poor ignoramus ; perhaps a hundred teeth, plugged so as to be effectually preserved, would not, in one year, counteract the effects of this bad operation.

But I take it, there are a great many reasons which operate to produce this want of faith, and indeed they are, in many places, formidable ones, for scarce a countervailing evidence exists, to eradicate the influence exerted by bad operations. Yet I think that all can be satisfactorily explained and accounted for, when we take into consideration the many errors which prevail in relation to dentistry. Let us, then, take a hasty glance at some of these errors, and, if possible, arrive at a remedy. I know not that these errors are as numerous in the east as in the west, yet I doubt not you have much error to contend with, more than is in any way desirable in practice ; I shall speak, however, more in reference to the actual state of things in the west.

I think I have somewhere seen a book, styled every man his own doctor. How often do we see this principle acted upon, by those who have never studied medicine. They are continually attempting to doctor themselves, while those who have studied most, scarcely ever do so in dentistry. Scarce a sufferer from decayed teeth or toothache, but has a remedy for both. They can trace the cause of decay from one stage to another—point out the phenomena of disease—dilate largely on the sharp, the dull, the heavy and the jumping toothache—tell terrible tales of swelling and bursting of the teeth—and wind up with the perfect charm-like effect of their remedy. In their theorising, they entirely forget the natural law of cause and effect, the laws of chemical affinity, and the difference between the dead and living tissue; all this matters not, however, to them—their happy state of ignorance shields them from any mortification which they might otherwise endure.

An accurate history of all the nostrums which have been recommended for the cure of toothache alone, would fill volumes, and unfold a mass of ignorance and superstition, perfectly humiliating to look upon. Superstition is too deeply rooted, and has too strong a hold on the feelings and prejudices of the mass of mankind, to be eradicated, in the natural advance of science, in one or two generations; nothing but the continued dissemination of truth, yea! all potent truth, before which error must vanish, can ever affect this great object. Medical science, in all its various departments, lay for centuries enveloped in the mantle of superstition; the labored researches of the great and wise have done much to cast off this garment of shame; and the study of man, the great masterpiece of a wondrous creation, in all his varied organization, with all the phenomena of healthy and diseased action, has become—save one—the most important which can engage our attention. How important, then, that this study be stripped of all the errors which naturally retard the advancement of science, and that the mind remain perfectly untrammelled in the reception of that truth which shall be elicited in our investigations.

I might name a fondness for that which is marvellous, as one of the errors which prevail to an alarming extent; this induces a susceptibility to a disease, with us called humbug, an epidemic, (if I may so define it,) which has prevailed for a long time, and, although generally not very fatal of itself, yet lays the system liable to more serious forms of disease, which often terminate in death. The patient, laboring under this disease regards but little the dictates of sound reason, but rather, like the inebriate with his bowl, becomes fond of it, and, notwithstanding all he suffers, as soon as convalescent, exposes himself to a relapse. But as this is more a disease than an error, I will resume the direct subject of my address.

The first error I shall notice, is more particularly confined to our profession. It is an impression that it requires no study, and but little mechanical education, to prepare the student for the practice of dentistry; the great desideratum appears to be mechanical genius. Perhaps, of all the errors which operate against the advancement of dental science, this is the most appalling; for, so long as this notion prevails, young men will not devote that time and study which is important in the preparation for practice. This error is sustained, too, by many who have acquired some reputation as dentists; who forget or disregard the honor and interest of their profession, for the sake of a small fee, and, to be consistent, labor to make their students believe they are adepts in the dental art.

Thus after laboring hard for years to arrive at the eminence they have attained, they go to work and do all in their power, indirectly, to demolish the superstructure they have sought to erect. This is no idle imagination, for it occurs daily around us, and the country is flooded with dentists of a few weeks' preparation. These again take students; and not knowing quite as much as their preceptor, and perhaps having an exalted opinion of their talents for teaching, or feeling that two or three weeks is sufficient to learn what they know, they at once establish this as the term of tuition. So long as this continues, our profession will never be deficient in the number who claim to compose the fraternity.

You are met at every turn with the assertion that any man can plug a tooth. Neighbor such-and-such-an-one extracts teeth; and by-the-by he pulls well too, for he holds on with a death grip, and pulled me three times around the room, and broke my jaw too, but he brought the tooth. This is the estimate many place on dental operations, and is often induced by that utter disregard of correct principles shown by many who claim to be dentists. The operation for cataract is a nice and delicate operation; no one would think of employing an incompetent occulist to operate on his eye—yet I venture to assert, that any young man of ordinary talent can sooner learn to excel in that operation, than in the plugging or even extracting of teeth. Let us then take the treatment of all opthalmic diseases, and make a distinct profession of it, as it really is.

Who would think of going into the office of an occulist, and learning his *trade*, as they call it, in a few weeks? Am I told, however, that this is not a just comparison. Why not? The operations strictly dental are more numerous, tedious, and difficult to perform than those properly opthalmic. But, says one, the diseases of the eye require medical treatment, and this cannot be done without a proper medical knowledge. I admit all this. But are not the dental organs also subject to disease, and equally require medical treatment? There is only this difference—we have thirty-two teeth and only two eyes, and, unfortunately for dentistry, the thirty-two teeth can be more easily parted with than the two eyes. A man will make some effort to preserve his organs of vision; when, at the same time, he will regard it as wrong—yes, as positively sinful, to attempt to preserve his organs of mastication. Both constitute an important part of the system—both are subject to disease, which exerts directly or sympathetically an influence on the general organization—each performs an important function, which cannot be dispensed with without curtailing the pleasure or endangering the health of an individual.

I never could find out what the teeth had done that they should be so neglected. Some appear to dislike the idea of having any thing as a part of their system of so hard a texture, for, from the earliest period of life, they studiously avoid all effort to preserve them. They take some care of their face and hands, but their teeth—O they must take care of themselves. Hence they soon fall a prey to utter negligence.

If diseases of the eye affect the general system, and show the importance of a knowledge of that system, the diseases of the teeth exert equally as pernicious an effect, and demonstrate the same fact. Indeed far more injury is caused to the general constitution by dental irritation than by all the opthalmic diseases which prevail—disease of the latter is often induced by disease of the former; hundreds, perhaps thousands, suffer from dental disease where one suffers from disease of the eye. If the relative merits of any profession was valued by the amount of benefit derived from it, none would hold a more important rank in the estimation of the public than that of dentistry. Every year I am more and more convinced, that the influence of dental irritation on the system, and the effect thus produced on other diseases which *affect that system*, is not, by any means, properly appreciated.

One of our most celebrated western occulists has become so well convinced of this fact, that almost invariably he examines the condition of the mouth before any general course of treatment is adopted. He contends, very properly, that all cause of irritation must be removed, before any permanent benefit can be expected from the use of medicine. The importance of this will be easily appreciated, when we recollect that the fifth pair of nerves, or trifacial, sends off at the ganglion of Casser three branches—one to the eye, and one each to the superior and inferior maxillary bones. Thus distributing to the eye, teeth, tongue, and, indeed, the entire face, an expansion of nerves intimately connected, and sympathising with each other in every attack of disease.

Another error which I shall notice, and which prevails to a considerable extent, is, that cheap materials will answer as well, and preserve the teeth as long as pure gold.

Unfortunately, this error also receives countenance from many who claim to be dentists. Cases do occasionally occur when a cheaper material than gold may be used without any censure to the dentist, but never when recommended as equal. Many persons, not able to have their teeth filled with gold, would do well to have them stopped with tin. But there are other materials which, notwithstanding the great efforts of this society, and I

believe well informed dentists everywhere, to prevent being used in the plugging of teeth, yet is used, and recommended by hundreds; I allude to mineral paste. It appears, that of late a regular itinerating business of paste-stuffing has sprung up throughout the length and breadth of this land. Go where you will, and whatever town or village you may enter, there you will find the track of this pestilence. Scarce a neighborhood but has been dosed with the royal succedaneum, and have suffered, and are still suffering from its pernicious effects. The very able report by Dr. Westcott, published in the fourth volume of the "American Journal and Library of Dental Science," preclude the necessity of saying much on this subject. The "Mississippi Valley Association of Dental Surgeons," at their organization last August, seeing the extent of this evil, and feeling the importance of expressing an opinion on the subject, passed, unanimously, the following resolution:

"*Resolved*, That we consider the use of all mineral paste, or other paste, in the plugging of teeth, as unprofessional and highly injurious, and that we will neither use it, nor countenance its use by others."

That a paste may ultimately be made, other than a compound of minerals, which may answer the purpose, I am not able to determine. When this does take place, and it answers the great purpose of preserving the teeth, I shall, for one, hail it as an epoch in dentistry, which will soon mark with perfection our art. Let this be accomplished, and so made as to adhere to the tooth, possessing proper color, and capable of being enamelled, and it would lead to results in our profession scarce less than the introduction of steam on our mighty waters. The pure gold is an article, however, which possesses all the requisite qualities for the plugging of teeth which we may expect from any mineral substance, and when properly used, must meet every reasonable expectation of the patient.

But, Mr. President, there are not only errors in relation to the use of materials for the filling of teeth, but errors also prevail in relation to the utility of certain operations, one of which I shall particularly notice. One tells us his teeth were ruined by the use of the file, another by cleaning, and the third lays it all on the use of calomel. That teeth may be injured by all these methods, I am free to confess; but not to that extent, or in the manner generally supposed. Let us first examine the objections to the use of the file, and see if they are well founded.

In using the file, the first object to be attained is the separation of the teeth, so as to expose the decayed surface. I take it for granted that no man is so fond of this operation as to file apart sound teeth. It would be just as rational to take medicine when well, to prevent sickness. But there is a disease, and our object is to get rid of it, and at the same time, as much as possible, prevent its recurrence. I know of but two ways to effect this—one by plugging, and the other by filing and removing the disease with such other instruments as are useful for that purpose. When the teeth can be filled, this, of course, is the operation which should be performed. But there are cases where this is impracticable, and here I contend, the use of the file is proper. It is also almost indispensable in the preparation of teeth decayed between, for plugging; and here, perhaps, more such operations fail because of the inefficient use of the file than from any other cause—for the cavity must be well exposed, or it cannot be properly filled. But the question recurs—will the removal of the decayed part (when not sufficiently deep to admit of a plug) arrest the further progress of the disease? I conceive the presence of a cavity in a tooth, whether from decay or otherwise, always exposes the part to the causes which bring on decay; hence, we find, that in the natural indentations and depressions of the teeth, disease generally first seats itself. This indeed forms the very basis of the present generally received theory of decay. Experience, however, proves beyond a doubt the utility of this operation. Hundreds of cases can be seen in almost every city, where teeth have thus been preserved for twenty, thirty, and even fifty years. But, says the objector, you file away the sound part of the tooth. In reply, I say, I file in no farther than the disease has penetrated, and remove that part only which can never preserve the tooth; but which, indeed, retains, as it were, the disease, and gives it opportunity to act on the sound part of that organ. The portion of the tooth removed, other than that already decomposed, is the enamel, which had once covered healthy bone. This diseased mass now requires no such covering, except, indeed, to retain the agent which promotes the decay. I cannot conceive any action of the file which would aggravate the disease already existing. If the action of the decayed portion of the tooth is more pernicious to the living substance than the air we breathe, and the fluids of the mouth, it is certainly important that it should be removed. Whether we consider this disease a caries or gangrene, or whatever term we may use to designate it, it is, at least, the destruction of the vital principle, which keeps, as it were, in a state of cohesion the bony structure. The vitality may be destroyed in the tooth, and yet this cohesion remain; yet this is always more or less destroyed where decay takes place.

The surgeon hesitates not to remove with his knife a cancerous tumor; and, although seated in that part of the system possessing, more or less, the power of recuperation, yet experience has shown that the most certain and effectual method of cure is the entire extirpation of the disease. He, therefore, cuts not off merely the surface of the disease, and leaves the vis medicatrix naturæ to accomplish the balance—no! he boldly cuts down, and aims at the entire removal of the dangerous disease. Thus would I act if I wished to arrest the decay of the teeth by the use of the file. When thus used, and at the proper time, a more effectual method of preserving the teeth is not within our reach.

I shall not attempt to describe the method of using the file, for I am merely speaking of those errors which prevail in relation to dental practice, and have more particular reference to that prejudice which exists in the public mind on this subject. There is no prejudice which is so often brought to bear on our operations as this. I have known the most intelligent persons object to the use of the file on their children's teeth, when those of their children were present whose teeth had been for *years*, and were still preserved by *this operation*—yes, object with this fact daily before them.

An individual calls on a dentist just when his teeth have commenced to decay; the disease has only made its appearance between the front incisors. These he properly separates, and leaves the filed surfaces so that they cannot come in contact. This is the last he sees of his patient for two or three years. In the meantime his other teeth have commenced to decay between the front and lateral incisors, canines, bicuspides, &c. He lets it progress until perhaps too far gone to be arrested by any means of our art. He then goes to another dentist, and wants something done, and has a long tale to tell of the miserable operation before performed, and contends it was the cause of the loss of all his teeth; when at the same time the best teeth he has are the two that were filed. I am not merely drawing on the imagination for a case, but have given the history of thousands that occur.

In 1831, I had the pleasure of performing a small operation for an intelligent young lady, near the city where I now reside. The operation was confined to the plugging of a molar tooth, and the separation of the front and lateral incisors, which had commenced to decay. Twelve years rolled around before I again had an opportunity of examining her teeth. I flattered myself that I had some hold on the kind regard of this fair lady. What think you was the salutation after this long absence? It was this—Doctor I have laid up for you a dreadful scolding—for at such a time you filed my teeth, and ruined the whole of them. With some hesitation, she permitted me to examine her teeth, and truly a lamentable devastation I witnessed. Scarce a sound tooth had she in her mouth—all the front teeth were gone, save half of the front incisor which had been filed; the molar, which I plugged, was the best tooth in her mouth; and on inquiry, I found the teeth that had been filed were lost, not from that operation, but from the disease which had attacked the adjoining teeth; and this was made apparent from the filed half of the front incisor which remained; and yet she labored under the belief that the separation of the two teeth had been the cause of the loss of all her teeth.

This case, when properly investigated, showed the utility of my operation, and conclusively proves that with proper care, her teeth might have been preserved. The operation had accomplished all that any operation of the kind could accomplish, but had not done all she expected—for she thought it should also preserve the other teeth.

From the great number of similar cases which occur, and that too among the more intelligent, I have come to the conclusion, that an individual may be well informed on general subjects, and yet vastly ignorant as it regards his teeth.

This is not as it should be—for no part of the system requires more care to preserve, but if properly attended to, no part is more certain of preservation. Does it not then become us as members of the dental profession to change, as it were, public sentiment, and diffuse that knowledge which is so essential to the preservation of these organs.

The notion which prevails to some extent in relation to the cleaning of teeth, scarce deserves a particular notice. Some savages think it great folly to wash their face and hands; they even plaster on a coating of oil and dirt to add beauty to their person and shelter themselves from the attack of gnats, and other insects, which abound in their country.

Tastes you know, vary, and fashions change with the season. Some have a taste for dirty teeth, and like that fashion best which affords least labor. At present, I shall not quarrel with such on this subject.

The next error to be attended to, is that which prevails, as it regards the effect of calomel (the sub-muriate of mercury) on the teeth. Perhaps any thing when taken into the mouth, and suffered to remain sufficiently long, which has a greater affinity for lime than the phosphoric acid, and not in combination with a base, for which it has a still greater affinity, would exert its chemical action, and decompose, as it were, the structure of the tooth. None who regard the cause of decay as a local chemical agent would deny this—for our treatment of this disease in all our operations is based on this hypothesis.

There is no notion much more prevalent in the community than that a few doses of this medicine will bring on decay; and although this opinion is so often expressed, and is continually made to ring in the ears of every dentist, yet I have never seen in any of our standard dental works a thorough investigation of this subject. Most, indeed, pass it over in silence. Many dentists think it highly injurious, and, like sulphuric acid, should be taken through a quill.

I grant that this medicine often does produce a disease more to be dreaded than decay, yet this is generally from an improper use. The best gifts of a bountiful Creator are sometimes abused. Let us remember that reason was given us that we might use the bounties of Providence in a rational manner, and thus even make the most virulent poisons subservient to our use.

Let us, however, first examine the chemical action of this agent on the teeth, when taken into the mouth. The structure of the teeth consists in the union of about forty-eight parts phosphoric acid, and fifty-one lime, with some animal matter. Thus it may properly be considered a sub-phosphate of lime. It is this substance which forms the basis of animal bone.

The sub-muriate of mercury contains to the 100 parts mercury, 13.99 muriatic acid, and 4.16 oxygen; the mercury very largely forming the base, which in its pure state is tasteless, and has no effect whatever on the teeth, it is, therefore, only in its union with oxygen, and some acid, that a chemical action could be expected; and unless that acid should have a greater affinity for lime than the phosphoric, and the latter more affinity for the mercury than the former, no such action could take place.

This we find to be directly the reverse, for the muriatic acid and mercury have a greater affinity for each other than the phosphoric and the mercury, and the phosphoric acid and lime unite more readily than the muriatic and lime. So, according to the laws of chemical affinity, there is no danger of the calomel exerting an injurious effect, chemically, upon the teeth. If it was not for this law of affinity, the muriate of soda (common table salt) would be still more pernicious, for this contains, according to the analysis of Dr. Marcet, forty-six parts muriatic acid and fifty-four soda. So here is over three times as much acid in a given quantity of table salt, to act upon and destroy the teeth, as in calomel. But here again chemical affinity interposes to save the teeth.

The following acids have a greater affinity for lime than the phosphoric, and should be used about the teeth with a great deal of caution—the oxalic, the sulphuric, the tartaric, and succinic; and yet I have known quantities of the dilute sulphuric put up in bottles, and sold through the country as a wash to cleanse and preserve the teeth. Perhaps I may have not given the accurate comparative affinities of these articles; but, more strictly speaking, I have given the actual order of decomposition. Heat will, to some extent, exert an influence, and somewhat affect the action of acids on these bodies. What effect will the vitality of the teeth have in resisting the action of these agents? or will it, in any measure, lay these organs more in danger of decomposition? I cannot think the latter. These are certainly questions, however, that much concern every dental practitioner; and I hope will draw forth much investigation in this department of our science.

But, am I asked, how is all this reconciled with the fact, that in the analyses of the teeth, muriatic acid is used? I answer, that although used, it is not in connection with a base for which it has a greater affinity than for the lime, for if so, it would in no case act as a solvent on the teeth. I am sorry that I did not commence a series of experiments early enough to give the result at this time. We know that the fluids of the mouth are frequently in a vitiated condition, sometimes produced by a disordered stomach, and sometimes by a disordered condition of the mouth itself; that this vitiated condition of the saliva exerts an injurious effect on the teeth, perhaps none would doubt. I hope, by repeated experiments, and a correct analysis of the saliva in a diseased and also healthy condition, to arrive at the cause of decay. The great difficulty in all our experiments arises from the fact that they have to be made, not on the living but dead structure.

The action of calomel on the system has been accounted for in two or three ways. There is first the mechanical hypothesis, contended for by Atrue, who fancied it to act by its weight, its divisibility and mobility, "thus getting into the blood, and rendering it more fluid and fit for secretion."

And second, there is the chemical hypothesis. Thus, Métié, Pressavin and Swediaur assumed, that it acted on the poison of some diseases as acids and alkalies on each other. Then we have the dynamical hypothesis; some contending for it as a sedative—some as a tonic—some as a stimulant, and some as an alterative. Our main object, however, is to ascertain what influence it exerts on the secretions of the mouth, and see if it can thereby affect the teeth.

According to Dr. Thompson's analysis, saliva, during mercurial salivation, contained, water 99.286, chloride of sodium 0.090, and the remaining fraction, mucus and albumen; so that only a very small quantity of chlorine can be found in this fluid, even under the most favorable circumstances. In the most careful analysis yet made, only a very small portion has ever been detected, not more, I believe, in the saliva than in almost any other part of the system. Thus, from the best evidence we have on the subject, calomel does not act chemically on the teeth, either locally, or through the secretions of the mouth. That this remedy sometimes acts very powerfully on the absorbent system, none will deny; and when this is the case, very injurious results may take place, to the dental organs, or more particularly to the periosteum and the soft parts of the mouth; for perhaps there is only one period in life, when it could even then exert a pernicious effect on the structure of the tooth, and that would be while the teeth are in process of formation. According to Rousseau and others, inflammation in the parts concerned in this process, during the development of the teeth, will show itself in erosions of that organ. But this only affects that portion formed during the action of this medicine, or during the disease, and, consequently, salivation would only affect that portion of the tooth formed during the action of this remedy. It seems very probable, that, during a severe salivation, the secreting vessels which form the teeth, can be so far divested from natural and healthy action, as to perform but imperfectly their appropriate function. This is a subject which I think yet requires a great deal of investigation, and is of great importance to our profession.

I think it a very great desideratum, to know the particular action of all the various remedial agents on the teeth; in this way alone may we expect to arrive at the true cause of decay, and also the proper treatment by which to prevent it. There are so many causes to modify or excite the action of chemical agents on the teeth in the mouth, which do not operate on inert matter, and out of the mouth, that what may affect a tooth in the mouth of an individual, would not out of it, and *vice versa;* so that, what would destroy a tooth out of the mouth, would, perhaps, in the living system, exert no pernicious influence.

I did intend to have said something on the effect of mercury as one of the exciting causes of that most troublesome disease, alveolar absorption, than which there is none other which causes the dental practitioner more trouble. But I find, Mr. President, that to do justice to the subject would have required more time than I had to devote to the subject, and would occupy, of itself, at least one address before this society. Besides, I set out with the intent of speaking of the various errors which prevail in relation to dental practice; and I find to enumerate the whole would make a short address, and at the same time afford a gloomy picture to dwell upon.

Yet permit me, briefly, to allude to another error, which, perhaps, does as much as any other, to make business for the great numbers which crowd our profession. This is the idea that when the nerve is once destroyed, there is a complete relief thereafter to the toothache; hence the various nostrums daily in use for that purpose. Perhaps there is no disease for which so many specifics have been discovered. Scarce an eight by ten sheet published in these United States, but contains one or more remedies for this horrible disease, generally headed, an infallible remedy for the toothache—an effectual cure for do.—the only specific ever yet offered to the public, for that distressing complaint, the toothache; and the pain is sometimes not only to be relieved, but the tooth is thus to be preserved and rendered useful for life.

I recollect of seeing, a few years since, in a London medical journal, nitric acid recommended as an effectual remedy, and it would not only relieve the dreadful pain in the tooth, but also arrest the further decay of the tooth. Would we be safe in recommending this article as a wash to preserve the teeth? for if it will arrest the decay, it will certainly prevent it. This would at once put a stop to dental operations, and turn us all, gentlemen, on the world, to hunt our living in some other avocation. Let it not be said, that we will destroy our profession in this way. I hope we are all too fond of our science, to thus at once blot it out of existence by a bottle of nitric acid.

It would be, perhaps, difficult to estimate the injury the public sustain by the use of quack nostrums for this disease; hundreds, yea thousands, sacrifice many of their best teeth, to retain a little longer an old root, or, what is equally bad, the shell of an old tooth, a fit receptacle for all the extra food to undergo decomposition in. I must confess I have no faith in odontalgic nostrums. When necessary to treat this disease at all, other than by extraction, it should be treated according to the strict principles of dental pathology. I know not of any subject on which grosser ignorance prevails, than that of toothache. Almost every thing, from the incantations of the dental conjuror to the scientific passes of the animal magnetizer, has been brought into requisition for the cure of this disease. He who would expect to cure periodentitis, by the same remedy as would relieve an inflamed dental nerve, certainly shows very little knowledge of the parts involved in disease; one is easily relieved by almost any remedy having the least pretensions to specific; the other will not be relieved by all the nostrums which are said to never fail in giving relief; one is an irritable condition of an exposed dental nerve, where an application can be made directly to the affected part; the other is an inflamed condition of a delicate membrane within the alveolar socket, and beyond the reach, to any extent, of any local remedy we can use. One is really a case in which to test the puffed nostrums of the ignorant pretender; the other gives celebrity to the veriest infinitesimal dose of the homœopathist. On the whole catalogue of remedies, thus put forth to relieve this disease, might be written the celebrated inscription, which so much startled the Babylonian monarch, "Thou art weighed in the balance, and found wanting."

But there are other cases often occurring, and somewhat characteristic of the age, if you please, and where no medicine is employed, possessing this advantage over all the others. If it does no good, it will do no harm—a very powerful argument in the minds of old women. Those fine and delicate cords which convey, throughout the system, the sense of feeling, often become morbidly alive to every impression; they vibrate under the gentlest touch of the morning breeze. The slightest tremor sets in motion a discord more jarring than that produced by the fall of the mighty tower which confused all language. A cloud obscures the sun, and refreshes the earth with the dews of heaven, but sends coursing on these magnetic rods a superabundant load of electric fluid.

The fifth pair of nerves, with their dental branches, terminating in a more solid structure, and less disposed to let this fluid circulate than the softer parts of the system, become, as it were, the galvanic battery, and set up a terrible action in the masticating organs of poor frail humanity. Well is it for mankind that a calm succeeds every storm that nature, in her laws which govern the vast machinery of this universe, seeks to restore her lost equilibrium. That for every positive, we have a negative pole; so that this all-pervading fluid soon becomes properly distributed throughout animate and inanimate nature. But should nature move too slow, and this battery keep up too long her shocks on the dental organs, soon (perhaps drawn hither by the laws of magnetic attraction) stands one of nature's *regulators*, and impelled by that same mysterious power, his arms commence to move, and with the gravity of the judge, when about to pronounce the awful sentence of the law on some poor criminal, he solemnly, but gently waves off the superabundant fluid. The nerves sink to repose beneath the downward move of this most potent agent; and, as if fanned by ambrosial zephyrs, the patient calmly and sweetly falls into the arms of morpheus. Here, says one, is a remedy more efficacious than all your drugs, and based on the most scientific principles which govern the animal economy. Truly a new era has dawned upon medical and dental science, and the seventh son no longer holds in his hands the charm by which disease and pain are held at abeyance. Even he who extracts the toothache by an operation on the ear stands appalled; his bloody knife drops from his hand; and soon the race of marked bipeds, like the Indians of the west, will disappear from our midst, and the magnetic doctor stand alone in his glory.

In what an age we live. The sluggish onward trot of steam's propelling power suits not this go-ahead generation. But with the lightning's flash, they must press on to greatness; beneath, above, around, and pressing in at every pore, an agent stands ready for our bidding; as quick as thought, it carries thought through illimitable space, and reports, with truthful fidelity, every word and sentence, with one foot on the western shore of the Atlantic, while with the other ready to stride the mighty ocean, and land with the latest news instanter in London. Scarce can you count the ticks which make up a moment of time, before the circuit of the earth is done, and at your side the faithful messenger is ready for another journey. This all-pervading mighty fluid, invisible, revivifying and electric agent of Him who "rides upon the whirlwind, and directs the storm;" that lights with a lurid flame the ethereal space, as He who speaks in the mighty thunder, directs its course, yields passively to the guidance of enlightened science.

In conclusion, permit me to say, that we of the west look to the proceedings of this society, at the present time, with more than ordinary interest. The success of a similar organization with us depends, in some measure, on the proceedings of this society. For if the national association maintains its dignity of character, and encircles, within its wide expanded arms, none but the worthy and meritorious, the Mississippi Association will find it very easy to follow the example of so distinguished a body of brotherhood.

